# Potential of Yeasts as Biocontrol Agents of the Phytopathogen Causing Cacao *Witches’ Broom Disease*: Is Microbial Warfare a Solution?

**DOI:** 10.3389/fmicb.2019.01766

**Published:** 2019-07-31

**Authors:** Pedro Ferraz, Fernanda Cássio, Cândida Lucas

**Affiliations:** ^1^Institute of Science and Innovation for Bio-Sustainability, University of Minho, Braga, Portugal; ^2^Centre of Molecular and Environmental Biology, University of Minho, Braga, Portugal

**Keywords:** Witches’ Broom Disease, Moniliophthora perniciosa, yeasts, biocide, antagonism, sustainability, cacao, phytopathogen

## Abstract

Plant diseases caused by fungal pathogens are responsible for major crop losses worldwide, with a significant socio-economic impact on the life of millions of people who depend on agriculture-exclusive economy. This is the case of the *Witches’ Broom Disease* (WBD) affecting cacao plant and fruit in South and Central America. The severity and extent of this disease is prospected to impact the growing global chocolate market in a few decades. WBD is caused by the basidiomycete fungus *Moniliophthora perniciosa*. The methods used to contain the fungus mainly rely on chemical fungicides, such as copper-based compounds or azoles. Not only are these highly ineffective, but also their utilization is increasingly restricted by the cacao industry, in part because it promotes fungal resistance, in part related to consumers’ health concerns and environmental awareness. Therefore, the disease is being currently tentatively controlled through phytosanitary pruning, although the full removal of infected plant material is impossible and the fungus maintains persistent inoculum in the soil, or using an endophytic fungal parasite of *Moniliophthora perniciosa* which production is not sustainable. The growth of *Moniliophthora perniciosa* was reported as being antagonized *in vitro* by some yeasts, which suggests that they could be used as biological control agents, suppressing the fungus multiplication and containing its spread. Concurrently, some yeast-based products are used in the protection of fruits from postharvest fungal spoilage, and the extension of diverse food products shelf-life. These successful applications suggest that yeasts can be regarded a serious alternative also in the pre-harvest management of WBD and other fungal plant diseases. Yeasts’ GRAS (Generally Recognized as Safe) nature adds to their appropriateness for field application, not raising major ecological concerns as do the present more aggressive approaches. Importantly, mitigating WBD, in a sustainable manner, would predictably have a high socioeconomic impact, contributing to diminish poverty in the cacao-producing rural communities severely affected by the disease. This review discusses the importance/advantages and the challenges that such a strategy would have for WBD containment, and presents the available information on the molecular and cellular mechanisms underlying fungi antagonism by yeasts.

## Introduction

A great number of plant diseases are responsible for major crop losses with huge socio-economic impact, causing each year a worldwide estimated losses of 40 billion dollars (Syed Ab Rahman et al., 2018). Particularly, the diseases caused by fungal pathogens are increasingly recognized as a global threat to food production and security. In fact, since 2000 the number of new fungal plant pathogen alerts has increased by more than sevenfold (Fisher et al., 2012). Presently, fungal-generated diseases constitute 64–67% of the total crop diseases reported globally (Fisher et al., 2012; Fisher et al., 2018), and account for 20% of the losses at the level of production and a further 10% at postharvest level (Fisher et al., 2018). These authors estimate that fungal diseases are spreading northbound at a rate of almost 8 km/year. This could derive from increasingly common agricultural practices, such as the extensive monocultures and the use of a restricted number of plant cultivars, as well as the increased global international trade proportionating disease spreading over great distances (Fisher et al., 2012). Climate change adds a burden to that equation, potentiating the development of microbes and vectors in unprecedented regions (Robert et al., 2015; Fisher et al., 2018).

To prevent plant diseases and protect crops from pests and pathogens, widely spread methodologies mainly correspond to applying chemical fungicides. The continued use of chemical fungicides leads to the development of fungicide resistance in the fungal pathogen (Syed Ab Rahman et al., 2018) and, in the absence of other control measures, to the re-emergence of virulence (Fisher et al., 2018). Therefore, in spite that the use of pesticides brought clear improvements in crop quality and quantity during more than half a century, their progressive inefficacy to treat some of the most harmful plant diseases requires the utilization of higher dosages each year (Medeiros et al., 2010; Syed Ab Rahman et al., 2018). The use of fungicides heavily impacts on the microflora of agrarian ecosystems, destroying beneficial microbes, such as endophytic bacteria and fungi, as well as animals important for the quality of the soils (Syed Ab Rahman et al., 2018). Ultimately, the systemic use of these drugs leads to the persistence of chemical residues in the environment, proportionating low dosage toxicity and contaminating species across trophic levels (Carvalho, 2006; Dukare et al., 2018).

**FIGURE 1:**
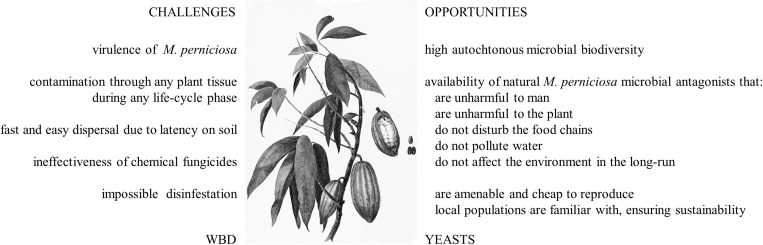
Diagram presenting the challenges posed by WBD and the opportunities that natural antagonistic yeasts present for the containment of the disease. Cacao plant figure adapted from Panhuys, L. von, Watercolours of Surinam (1811–1824) (source: (CC) BY-NC-ND http://plantillustrations. org/illustration.php?id_illustration=190533).

One of the fungal diseases with recognized high negative socio-economic impact is the pathology of cacao plant and fruit known as *Witches’ Broom Disease* (WBD). This is caused by the basidiomycete fungus *Moniliophthora perniciosa* (Aime and Phillips-Mora, 2005) (formerly designated *Crinipellis perniciosa*). The severity and extent of its manifestation is endangering the rapidly expanding and very quality-demanding chocolate market. This review focuses on this disease, the methodologies presently used for containment, and the innovative approaches that could be explored (Figure 1).

## *Witches’ Broom Disease* of Cacao

According to data from the International Cacao Organization^1^, more than 4 million tons of cacao beans are produced annually (Wickramasuriya and Dunwell, 2018). Cacao beans are the core raw material for the chocolate industry, although other cacao-derived products also have important world markets, such as cacao butter or liquor (Pohlan and Pérez, 2010; Wickramasuriya and Dunwell, 2018). The economic global market for chocolate reached US$ 110 billion in 2015, and the world demand is expected to grow exponentially in the next decade due to the globalization of consumption styles in expanding economies such as China and India (Squicciarini and Swinnen, 2016). Cacao is produced in countries located approximately in the same latitude interval of equatorial climate, forming the so-called Cacao Belt (Pohlan and Pérez, 2010). The biggest producers are therefore countries from Central and South America and Africa. The cacao plant is affected by several diseases, the more threatening of which is WBD (Purdy and Schmidt, 1996; Pereira, 1999; Griffith et al., 2003; Aime and Phillips-Mora, 2005; Teixeira et al., 2015). This has severely affected South and Central America countries, where it has been responsible for major irreversible crop losses. The highest economic and social consequences of WBD are described to have occurred in Brazil. In the 10 years after the onset of the disease in 1989, WBD reduced the cacao production in more than 70% (Pereira et al., 1989; Santos Filho et al., 1998; Trevizan and Marques, 2002; Meinhardt et al., 2008; Pires et al., 2009; Teixeira et al., 2015), causing Brazil to shift from being the 2nd world producer to becoming a net importer of cacao beans (Bowers et al., 2001; Marelli et al., 2009; Teixeira et al., 2015). During that period, the most affected region of Bahia suffered losses around 90%, configuring a severe social crisis from losing more than 200,000 farm jobs (Trevizan and Marques, 2002; Teixeira et al., 2015).

The severity of WBD derives from several factors. *Moniliophthora perniciosa* does not form specialized infection structures such as *appressoria* like other fungal pathogens. Since it is a hemibiotrophic fungus, the full infectious cycle unfolds through two distinct phases: (i) biotrophic and (ii) saprotrophic. (i) The initial infection occurs in young meristematic tissues and susceptible actively growing tissues (e.g., buds, young leaves, flower cushions, young fruits). The fungus penetrates through the stomatal openings, the bases of damaged trichomes and the husk of young fruits (Aime and Phillips-Mora, 2005). After the initial infection, the fungus induces hypertrophy and hyperplasia, causing the loss of apical dominance. This corresponds to a disorganized proliferation of the infected vegetative meristems of axillary shoots that results in the formation of a broom-like structure of abnormal stems called a *green broom* (Aime and Phillips-Mora, 2005; Meinhardt et al., 2008; Pires et al., 2009). Shortly after the initial infection, the fungus starts growing intercellularly, forming a monokaryotic and parasitic mycelium without clamp connections, establishing a biotrophic relationship with the host that corresponds to its life cycle biotrophic phase. (ii) Usually 4–6 weeks after the development of the *green brooms*, a concerted series of infected plant cells death events occurs and the infected tissues become necrotic forming a structure called *dry broom*. Necrotic or dead host cells are then colonized by the fungus (Evans, 1980; Meinhardt et al., 2008), which at this point, suffers major morphological changes entering its saprotrophic phase (Lawrence et al., 1991; Meinhardt et al., 2008). The hyphae become dikaryotic, clamp connections are formed, and the fungus begins to grow intracellularly, as well as between cells. The exact mechanisms and signaling factors that trigger the switch from the biotrophic phase to a saprotrophic phase, controlling the developmental alterations, remain unknown (Meinhardt et al., 2008). After the fungus proliferation and colonization of the dead host tissues, pink colored basidiocarps (small mushrooms) are produced on any infected necrotic tissue. Upon alternate wet and dry periods, each basidiocarp can produce 2–3.5 million spores (basidiospores), this way completing the fungus life cycle (Rocha and Wheeler, 1985; Almeida et al., 1997). The release of the spores occurs mainly at night, and is related to a high level of humidity and favorable temperature (20–30°C). The spores are disseminated locally by water and over long distances by wind, and can endure latent in the soil or inside pruned branches of the plants for long periods (Meinhardt et al., 2008; Pohlan and Pérez, 2010). The ability of *Moniliophthora perniciosa* to infect the plant in all stages of its life-cycle and the fact that virtually all the plant tissues can be infected, underlie this pest exceptional virulence. This, allied to the fungus high prevalence in the soil and plant dead material, explains why once a single plant develops symptoms the whole plantation can be compromised.

The resilience of *Moniliophthora perniciosa* relies essentially on its capacity to colonize both alive and dead plant tissue, the biotrophic and necrotrophic life cycle phases above mentioned. The shift between the two phases involves a drastic morphological and life style change. Alternative Oxidase (AOXp)-respiration was associated with this transition (Thomazella et al., 2012). Possibly, this type of respiration allows the fungal cell to overcome the plant host defenses generated in the first stages of the WBD, as observed with better studied model fungus *Ustilago maydis* (Cárdenas-Monroy et al., 2017). The plant defenses include the production of high amounts of NO, which affect the fungal mitochondria, namely inhibiting respiration complex IV, this way inducing the production of ROS (Thomazella et al., 2012). AOXp is an alternative mitochondrial oxidase that constitutes alone a bypass to respiratory chain complexes III and IV, which function prevents collapse from drugs that target these complexes like cyanide or Antimycin-A (Maxwell et al., 1999; Ruy et al., 2006; Vanlerberghe et al., 2009). At the same time, AOXp-respiration contributes to cope with the electron flux overflow without phosphorylation, and therefore without producing ATP (Van Aken et al., 2009) lowering the global energy yield of metabolism. AOX-encoding gene sequences are found in many organisms (including yeasts and fungi) (Elthon and McIntosh, 1987; Joseph-Horne et al., 2001; Stenmark and Nordlund, 2003; Chaudhuri et al., 2006; McDonald et al., 2009), but their physiological role in microbes is still not very well understood (Veiga et al., 2003; Rogov and Zvyagilskaya, 2015). In the case of *Moniliophthora perniciosa*, AOXp activity would prevent excessive ROS accumulation inside the fungal cell induced by the host, while maintaining a low metabolism status (Thomazella et al., 2012), which could explain how the fungal cell stays alive while the plant weakens.

In spite of the few *Moniliophthora perniciosa* genome surveys in the attempt to identify proteins/genes involved in this fungus infectious behavior (e.g., Mondego et al., 2008; Rincones et al., 2008), in fact little is known on this fungus molecular biology, mostly because as all non-model organisms it lacks the appropriate tools for genetic manipulation. Still, some physiological traits apart from AOX-respiration were shown in connection to this fungus pathosystem, like the increased secretion by the host of malondialdehyde (MDA) (usually a marker of oxidative stress; Del Rio et al., 2005) and glycerol (Scarpari et al., 2005), as well as of a methanol oxidase (MOX) (de Oliveira et al., 2012). Additionally, as for other fungal infections, also in this case the levels of the plant growth hormone ethylene are hypothesized to have a crucial role in the progression of the WBD, in particular in the development of the broom (Scarpari et al., 2005). The biological meaning of these findings remains unknown.

## *Witches’ Broom Disease* Management

The chemical fungicides generally used are either copper-based compounds, such as cuprous oxide, or azole-containing molecules, particularly tebuconazole (Oliveira and Luz, 2005; Medeiros et al., 2010). These are usually used to control the spread of other fungal plant diseases such as grapevine downy mildew and olive peacock spot, but showed very low efficiency against *Moniliophthora perniciosa* (Medeiros et al., 2010). Copper is *per se* a non-specific anti-microbial agent able to destroy naturally occurring microorganisms, including fungi, that is for decades applied as foliar sprays (Yang et al., 2011; Husak, 2015). The lethal action of copper-based fungicides derives from their ability to free copper ions that are massively internalized by the fungal cells. Intracellularly, they bind various chemical groups (imidazoles, phosphates, sulfhydryls, hydroxyls) namely in proteins, causing their denaturation and loss of function (Husak, 2015; Mirković et al., 2015). Ultimately, this leads to irreversible cell damage and membrane leakage (Husak, 2015). Yet some fungi are resistant to copper ions. The mechanisms underlying this resistance are not well understood, although several studies have suggested that they might exert a combined action: the extracellular chelation and cell wall sequester of copper ions, and their decreased intake and intracellular complexing by metallothioneins or other proteins (Cervantes and Gutierrez-Corona, 1994). This last case includes the over-expression of the superoxide dismutase (SOD) that uses copper as inorganic co-factor (Naiki, 1980). SOD has been described to display the ability to buffer copper excess independently of its superoxide scavenging function (Culotta et al., 1995). Additionally, the copper-induced accumulation of glycerol was also described to be involved in its extrusion (Gadd et al., 1984). Neither of these mechanisms were ever described in association with *Moniliophthora perniciosa*, although increased levels of SOD would contribute the higher resistance to the above-mentioned plant-generated ROS.

On the other hand, tebuconazole, as other azole-fungicides, acts on the synthesis of ergosterol, altering the structure and functionality of the fungal cell membrane (Price et al., 2015) as well as vacuolar ion homeostasis through v-ATPase function (Zhang V. Q. et al., 2010). In consequence of ergosterol synthesis disruption, mitochondrial function is also affected in its ability to form iron-sulfur clusters, which results in the deposit of insoluble iron inside mitochondria and concomitant radical formation and mitochondrial loss (Ward et al., 2018). CytC harbors a *heme* group which availability for this enzyme proper assembly and function would be affected by iron homeostasis disruption, consequently affecting Complex IV function in respiration. This would justify why fungi that can respire through the AOXp could be resistant to azoles. Still, there is no reference to this possibility in the literature. Rather, in *Candida* species, the resistance to azole-fungicides implicates other types of mechanisms (Whaley et al., 2017). Nevertheless, these are human commensals and pathogens, having therefore specificities that are not common with other yeasts or fungi.

Fungicides fail to control the spread of the WBD but the mechanisms underlying the resistance of *Moniliophthora perniciosa* to these drugs are not studied. The use of fungicides is therefore not a routine practice in most cacao-producing countries also due to their high cost, and the risks associated with cacao chemical contamination which hinders commercialization. The reasons underlying this include the increasingly considered negative impact of fungicides on human health and the environment. Public concerns regarding the prevalence of agronomic pesticide residues in food, and their relation with the increasing advent of pesticide resistant pathogens, not only in plants but also in humans (Droby, 2006; Pal and McSpadden Gardener, 2006; Marelli et al., 2009; Verweij et al., 2009; Nunes, 2012) led to restrictions in Europe. Therefore, the most commonly used methods to control the WBD are exclusively agronomic, through phytosanitary pruning, removing as much as possible the infected material, which is though often impossible, due to hidden fungal inoculum in the soil and cut branches and leaves^2^. Therefore, more effective and eco-friendly methods and strategies are needed to satisfy the consumer demands.

A concept that has gained considerable prominence in the agriculture sector in recent years is the use of nanoagroparticles, which are nanoparticles designed to mitigate agriculture-related problems, including plant pathologies (Parizi et al., 2014; Parisi et al., 2015). These nanoagroparticles include silver, copper, sulfur, zinc oxide and magnesium oxide nanoparticles (Baker et al., 2017), and can act efficiently as fungicides, pesticides, herbicides and also insecticides. They can easily enter into the fungal cell wall. Once inside the cell, they act through different modes, which include (i) causing the disruption of metabolism, or of the cell membrane, with consequent loss of cellular content (Baker et al., 2015, 2017), (ii) promoting the release of toxic ions (Cd^2+^, Zn^2+^, and Ag^+^) that bind to sulfur-containing proteins, (iii) targeting the pathogen DNA, this way inducing cell death, (iv) interrupting electron transport, this way causing the collapse of membrane potential, (v) promoting the generation of ROS, or (vi) interfering with nutrient uptake (Alghuthaymi et al., 2015). More than one of these mechanisms can occur simultaneously, conferring the ability of nanoparticles to be effective against different plant pathogens (Alghuthaymi et al., 2015). Recently, nanoparticles were conjugated with some biomolecules (including biocide/killer toxins), forming bionano-hybrid agroparticles (Baker et al., 2017). The free utilization of these promising phytopathology management tools still requires not only cytotoxicology studies to evaluate potential harm to human and animal health, but even more important, extensive ecotoxicology and biodegradability studies to evaluate their prevalence in the environment and food chains and their effect on the long run in the microflora of plant, soil and water. Presently few information on this regard is available (Alghuthaymi et al., 2015). Nonetheless, the prospective of being able to use such a nanotool to effectively deliver a toxin and kill a phytopathogenic fungus is attractive, especially if carrying a bio-derived killing agent.

## Yeasts as Biocontrol Agents

One possible approach to fungal diseases in plants might be the use of biocides or biological control agents. In phytopathology, this term designates the use of introduced or resident living organisms to contain or suppress populations of pathogens (Pal and McSpadden Gardener, 2006). There are a few of these agents in the market, mostly used in the control of pests at small scale. They correspond to dry biomass of bacterial or filamentous fungal strains isolated from the endosphere or the rhizosphere of plants (O’Brien, 2017) that are re-hydrated and used as alive reproductive microorganisms. These include a taxonomically and biologically diverse group of endophytic fungi that are characterized by colonizing internally the plant host tissues without causing any external disease symptoms (Wilson, 1995; Rubini et al., 2005). These endophytes can prevent pathogen infection and propagation directly by competition, mycoparasitism or antibiosis, or indirectly by inducing resistance responses in the plant (Bailey et al., 2006). Despite this, the biocontrol agents that could be applied in phytopathology are not restricted to these two groups of organisms.

Endophytic microorganisms also include Ascomycota and Basidiomycota yeasts, found in many species of trees from very diverse climates, but also in agricultural species (reviewed by Doty, 2013). Ascomycota yeasts reproduce exclusively by budding, as the most well-known yeast *Saccharomyces cerevisiae*. Basidiomycota grow dimorphically, shifting from a monokaryotic yeast-form to a dikaryotic filamentous form (Choudhary and Johri, 2009). This is the case of *Rhodotorula* and *Cryptococcus* sp. (Table 1). Generally, endophytic yeasts apparently thrive symbiotically or mutualistically, virtually colonizing diverse plant tissues (reviewed by Doty, 2013), in which they may cause structural changes (Luna, 2017). They consume sugars and assimilate amino acids generated by the plant, and contribute to the plant wellbeing and stress response in many different ways, including the production of phytopheromones, catalase or siderophores (reviewed by Joubert and Doty, 2018). Importantly, endophytic like epiphytic yeasts can antagonize phytopathogenic filamentous fungi, either by occupying their niche or by antagonizing them in more complex ways.

**TABLE 1:** Success cases of using yeasts to antagonize the spoilage of fruits by filamentous fungi.

**Yeast antagonist**	Host	Fungal phytopathogen(s)	**References**
Preharvest application^1^			
*Candida (Pichia) guilliermondii*	Cherry tomato	Fruit decay agents	Zhao et al., 2011
*Candida sake*	Apple	Penicillium expansum	Teixidó et al., 1999
Postharvest application			
*Aureobasidium pullulans*	Pear	Penicillium expansum	Robiglio et al., 2011
	Apple	*Botrytis cinerea*, *Colletotrichum acutatum* and Penicillium expansum	Mari et al., 2012
*Candida (Pichia) guilliermondii*	Chili	Colletotrichum capsici	Chanchaichaovivat et al., 2007
	Tomato	Rhizopus nigricans	Zhao et al., 2008
		Rhizopus stolonifer	Celis et al., 2014
	Kiwifruit	Botrytis cinerea	Sui and Liu, 2014
	Papaya	Colletotrichum gloeosporioides	Lima et al., 2013
*Candida oleophila*	Banana	*Colletotrichum musae*, *Fusarium moniliforme* and *Cephalosporium* sp.	Lassois et al., 2008
	Apple	*Penicillium expansum* and Botrytis cinerea	Liu et al., 2012
*Candida pelliculosa*	Tomato	Botrytis cinerea	Dal Bello et al., 2008
*Candida sake*	Apple	Penicillium expansum	Morales et al., 2008
*Cryptococcus infirmominiatus*	Sweet cherry	Monilinia fructicola	Spotts et al., 2002
*Cryptococcus laurentii*	Strawberry	Botrytis cinerea	Wei et al., 2014
	Sweet cherry	Fruit decay agents	Tian et al., 2004
*Debaryomyces hansenii*	Peach	Rhizopus stolonifer	Mandal et al., 2007
	Mandarin, orange	Penicillium digitatum	Taqarort et al., 2008
*Metschnikowia fructicola*	Apple	Penicillium expansum	Liu et al., 2011
	Grapefruit	Penicillium digitatum	Hershkovitz et al., 2013
*Meyerozyma caribbica*	Mango	Colletotrichum gloeosporioides	Bautista-Rosales et al., 2013
*Pichia membranefaciens*	Apple	*Monilinia fructicola, Penicillium expansum*, and Rhizopus stolonifer	Chan and Tian, 2005
*Rhodosporidium paludigenum*	Cherry tomato	Botrytis cinerea	Wang et al., 2010
*Rhodotorula mucilaginosa*	Pear	Penicillium expansum	Hu et al., 2015
*Rhodotorula rubra*	Tomato	Botrytis cinerea	Dal Bello et al., 2008
*Wickerhamomyces (Pichia) anomalus*	Banana	*Colletotrichum musae*, *Fusarium moniliforme* and *Cephalosporium* sp.	Lassois et al., 2008
	Orange	Penicillium digitatum	Aloui et al., 2015; Platania et al., 2012
	Papaya	Colletotrichum gloeosporioides	Lima et al., 2013
Commercial yeast-biocontrol products^2^			
*Aureobasidium pullulans*	Pome	Penicillium, Botrytis, Monilinia	Boni Protect^§^, Bio-Ferm, AT
*Candida oleophila*	Pome	Penicillium, Botrytis	Nexy^§^, Lesaffre, BE
*Metschnikowia fructicola*	Pome, table grape, stone fruits, strawberry, sweet potato	Penicillium, Botrytis, Rhizopus, Aspergillus	Shemer^§^, Bayer/Koppert, NL

*^1^Preharvest application to prevent postharvest spoilage. ^2^Wisniewski et al. (2016).*

The antagonistic interaction of yeasts with particular phytopathogenic fungi has been described in the literature. For example, Suzzi et al. (1995) observed that natural wine yeast strains of *Saccharomyces* and *Zygosaccharomyces* inhibited *in vitro* the growth of 10 species of soil-borne fungal plant pathogens, namely *Cladosporium variabile*, *Rhizoctonia fragariae*, *Phomopsis longicolla*, *Colletotrichum acutatum*, *Aspergillus niger*, *Sclerotinia sclerotiorum*, *Penicillium digitatum*, *Macrophomina phaseolina*, *Trichoderma viride*, and *Botrytis squamosa*. Also, Walker et al. (1995) reported that strains of *Saccharomyces cerevisiae* and *Pichia anomala* (know designated *Wickerhamomyces anomalus*) inhibited *in vitro* the growth of several wood decay basidiomycetes including *Serpula lacrymans*, *Postia placenta*, *Lentinus lepideus* and *Ophiostoma ulmi* and phytopathogenic fungi, such as *Rhizoctonia solani*, *Fusarium equiseti*, *Botrytis fabae*, and *Phytophthora infestans*. Importantly, Rosa-Magri et al. (2011) described the antagonism effect of the yeast *Torulaspora globosa* against the phytopathogenic mold *Colletotrichum graminicola*, the causal agent of anthracnose disease in maize. All of these cases were reported as *in vitro* studies, none were performed *in planta* or *in field*. Otherwise, yeasts have often been proposed and used for the control of microbial contaminations at the postharvest phase (Table 1).

The possibility of using yeasts as biocontrol agents of fungal or bacterial proliferation associated with food spoilage has been recognized since the early 1960s, when it was found that *Saccharomyces cerevisiae* strains secreted toxins that killed other yeast strains but are immune to their own toxin (Bevan and Makower, 1963). Killer toxins (KTs) can be encoded by cytoplasm-inherited double-stranded RNA viruses (Schmitt and Breinig, 2002) or linear dsDNA plasmids (Schaffrath and Meinhardt, 2005), but they can also be chromosomally encoded (Suzuki, 2005). The killer phenomenon is well characterized and studied in *Saccharomyces cerevisiae*. In this yeast species, KTs have been grouped into four types, K1, K2, K28, and Klus, based on their killing profiles and lack of cross-immunity (Schmitt and Breinig, 2006; Rodríguez-Cousiño et al., 2011). Each strain producing one specific toxin kills strains from the other groups but has self-protective immunity (Schmitt and Breinig, 2006).

The modes of action of the *Saccharomyces cerevisiae* KTs are well known, with the exception of the recently found Klus toxin (Schmitt and Breinig, 2002). K1 and K2 toxins kill sensitive yeast cells in a receptor-mediated two-step process. The first step involves a fast, energy-independent binding to a primary toxin receptor (R1), consisting of β-1,6-D-glucan (Lukša et al., 2015). Though a second energy-dependent step, the toxin is translocated from the cell wall to the plasma membrane, where it interacts with a secondary membrane receptor (R2), identified in the case of K1 toxin as Kre1p, an *O* -glycosylated protein of the yeast cell surface (Breinig et al., 2002, 2004). After reaching the plasma membrane, K1 and K2 toxins disrupt its function by forming cation-selective channels, and promoting the release of ATP and other metabolites, thus causing a lethal effect on the target cell (Liu et al., 2015). The K28 KT mode of action is very different, since it enters a sensitive target yeast cell by endocytosis, in a cell wall receptor-mediated manner (Schmitt and Breinig, 2006). The cell wall receptor for K28 toxin has been identified as a mannoprotein with high molecular mass (Liu et al., 2015). The K28 toxin is internalized through the secretory pathway (via Golgi and ER), and after entering the cytosol the β-subunit is ubiquitinated and degraded in the proteasome. The subsequently free small α-subunit has been suggested to enter the nucleus without the help of an active nuclear import machinery, therefore by a so-called passive diffusion (Schmitt and Breinig, 2006). Once inside the nucleus, the K28 toxin kills the host cell by irreversibly blocking the DNA synthesis. The target cells arrest in early S phase of the cell cycle, forming a medium-sized bud and a single, pre-replicated nucleus in the mother cell, eventually dying (Schmitt and Breinig, 2006).

The killer phenomenon, despite being best characterized in *Saccharomyces cerevisiae*, is not confined to this yeast species, rather it is often found in other yeast species and genera (Magliani et al., 1997; Schmitt and Breinig, 2002). Some of these were described to damage the plasma membrane, very similarly to the *Saccharomyces cerevisiae* K1 toxin. This is the case of the KTs produced by *Pichia kluyveri* (Ahmed et al., 1999), *Pichia membranifaciens* (Santos and Marquina, 2004; Santos et al., 2009), *Pichia farinosa* (Suzuki et al., 2001) and *Zygosaccharomyces bailii* (Weiler and Schmitt, 2003). Other killer mechanisms include the damage of the cell wall upon the inhibition of the synthesis of β-glucans. Examples of this mode of action are the toxins produced by *Hansenula mrakii* (previously *Williopsis mrakii*) (Marquina et al., 2002), *Wickerhamomyces anomalus* (formerly designated *Pichia anomala or Hansenula anomala*) (Wang et al., 2007), *Williopsis saturnus* (Guyard et al., 2002; Peng et al., 2010), and *Kluyveromyces phaffii* (Comitini et al., 2009). Yet other yeast KTs act by blocking the cell cycle, namely the one produced by *Kluyveromyces lactis* (Klassen et al., 2004), and by triggering DNA damaging and the induction of apoptosis, which is the case of the toxins secreted by *Pichia acaciae* (Klassen and Meinhardt, 2005) and *Wingea robertsiae* (Klassen and Meinhardt, 2002). The blocking of calcium uptake was also described as killer mode of action, namely in the case of *Ustilago maydis* (Gage et al., 2001). Although there are plenty of reports in the literature regarding the interaction of non-*Saccharomyces* killer yeasts with a vast variety of sensitive targets, the actual mechanisms involved remain mostly unknown or superficially studied at the molecular level.

Several other mechanisms of yeast antagonism have been proposed that do not involve the secretion of a peptide/protein that may be classified as a KT. Other proteins that are secreted by the yeast and antagonize filamentous fungi are lytic enzymes that destroy the fungal cell wall (Spadaro and Gullino, 2004). This kind of antagonism is considered a form of mycoparasitism. An example is the manner in which *Pichia guilliermondii* antagonizes *Botrytis cinerea* (Wisniewski et al., 1991). The authors observed that the fungus cell wall glucans and a yeast-secreted β-(1–3) glucanase form a lectin-like interaction resulting in a strong attachment of the antagonist to the fungal pathogen which culminates with the lysis of fungal cells. Yeasts that antagonize other yeasts can also produce several volatile compounds against filamentous fungi, like low molecular weight lipophilic compounds that inhibit the target growth (Mari et al., 2016). This is the case of the yeast *Aureobasidium pullulans* that produces 2-methyl-1-butanol, 3-methyl-1-butanol, 2-phenethyl alcohol and 2-methyl-1-propanol, with inhibitory effect against *Botrytis cinerea*, *Colletotrichum acutatum*, *Penicillium expansum*, *Penicillium digitatum*, and *Penicillium italicum* (Di Francesco et al., 2015). Interestingly, it appears that antagonizing yeasts may operate in different ways also because in some cases they strongly attach to the fungus hyphae. This was described in detail for the case of the yeasts *Pichia membranefaciens* and *Cryptococcus albidus* when challenged with three phytopathogenic fungi causing the postharvest deterioration of nectarines and apples (*Monilinia fructicola, Penicillium expansum*, and *Rhizopus stolonifer*) (Chan and Tian, 2005).

Another effective mechanism of antagonism and possibly the most common is competition. Microbes compete for space, for oxygen and of course for nutrients, such as carbohydrates, vitamins, minerals, and amino acids (Spadaro and Droby, 2016). Yeasts grow much faster than filamentous fungi, being thus able to quickly colonize the niches that fungi can occupy, such as plant wounds or tissue lesions, forming colonies or biofilms (Andrews et al., 1994). Increasingly bigger yeast populations reduce the amount of nutrients available for fungi and make them difficult to access (Zhang D. et al., 2010). In the case of micronutrients, iron plays a crucial role in the growth, development and virulence of the fungal pathogens (Saravanakumar et al., 2008). To compete with the pathogens for iron, yeasts secrete siderophores that deplete iron from the growth medium such as pulcherrimin, produced by *Metschnikowia pulcherrima* to compete with *Botrytis cinerea*, *Alternaria alternata*, and *Penicillium expansum* (Saravanakumar et al., 2008).

Ultimately, the efficiency of the antagonism is affected by environmental constraints (Syed Ab Rahman et al., 2018), and each mechanism of action depends on a specific interaction between the pathogen and the antagonizing organism but also between either and the host. It has been described that antagonizing yeasts may help the host by alleviating the production of ROS induced by the pathogen (Liu et al., 2013). The production of ROS is actually not confined to plants themselves, but also happens in fruits during postharvest processes. This was observed to occur when yeasts such as *Cryptococcus laurentii* and *Rhodotorula glutinis*, which exhibit antagonistic activity against the postharvest pathogens *Botrytis cinerea* and *Penicillium expansum*, were applied to fruit surfaces and wounds (Castoria et al., 2003). Concurrently, a biofilm of a *Saccharomyces cerevisiae* strain isolated from wine prevented the spoilage of apples by *Penicillium expansum* (Scherm et al., 2003). The potential of killer yeasts has often been recognized, but their use seldom put to practice for economically viable processes. In biotechnology, yeast-to-yeast antagonism has been mostly successfully applied in the control of wine and brewery processes, to avoid secondary fermentations producing undesirable compounds (Hatoum et al., 2012; Mehlomakulu et al., 2014; Oro et al., 2016). Another example of successfully commercialized application of a killer yeast is that of *Debaryomyces hansenii*, used in the prevention of spoilage of dairy products, such as milk and yogurt (Liu and Tsao, 2008), as well as in the extension of shelf-life of dry-cured meats and dry-fermented sausages (Andrade et al., 2014; Núñez et al., 2015). The spoilage prevention by yeasts extends to the postharvest protection of fruits from fungal spoilage, which acknowledges that yeasts can display significant antimycotic activity (Table 1). The majority of these antagonistic yeasts are naturally present on fruit and vegetable surfaces (Suzzi et al., 1995) but can be also obtained from other sources such as the phyllosphere (Kalogiannis et al., 2006), the rhizosphere (Long et al., 2005) or the soil (Zhao et al., 2012). The advantages of using naturally occurring yeasts strains are those of overcoming the negative effects of needed adaptation to the biophysical and biochemical specificities of the contaminated niches.

## Advantages and Challenges of Microbial Warfare

The use of live antagonist microorganisms or their nano-conjugated bio-derived toxins to manage phytopathogenic diseases would offer a great number of advantages over chemical fungicides or extensive pruning, including the safer field application methods and the reduced costs of production (Bonaterra et al., 2012), offering an environmentally friendly and bio-sustainable alternative to manage fungal diseases, provided the right microorganism is used. Any such option has to be primarily challenged with tests *in planta* and *in field.* Moreover, the solution, as mentioned above, has to be proven unharmful to human or animal cells (Ocampo-Suarez et al., 2017), as well as easily degradable so that its prevalence in the agriculture ecosystems does not generate a potential accumulation-derived harmful effect over time, and non-ecotoxic allowing the ecosystem functions to prevail (Liu et al., 2013). Finally, the commercial viability of any biocontrol agent needs to be subsequently assessed in pilot, semi-commercial and large-scale commercial studies, prior to regulatory licensing from the respective regulatory agencies (Droby et al., 2009). The final approval needs to be based on the actual disease control efficacy and also the evaluation of the safety of the formulated product (Dukare et al., 2018). For this reason, and despite the recognition of the efficiency of microbial antagonisms and the theoretical advantages of their use, only a limited number of products based on biocontrol agents have been formulated and commercialized over the past decades (Dukare et al., 2018). These products are registered for using mainly against some postharvest fungal phytopathogenic diseases of fruits and vegetables (Sharma et al., 2009; Sundh and Melin, 2011; Dukare et al., 2018). As an example, Nexy^®^ (Table 1) a formulation of water dispersed granules of living biomass of the yeast *Candida oleophila*, has been approved and commercialized in the EU against postharvest spoilage of stored fruits, namely against *Penicillium expansum* and *Botrytis cinerea* in apples, *Penicillium* spp. in citrus fruits and *Colletotrichum musae* in bananas (Ballet et al., 2016).

The microorganisms described to antagonize filamentous fungi include endophytic fungi, which have been suggested as biocontrol agents of several cacao plant diseases (Arnold et al., 2003). These authors demonstrated that inoculating cacao plant leaf tissues with fungal endophytes isolated from naturally infected asymptomatic hosts, significantly decreased the damage provoked by the foliar pathogen *Phytophthora* sp. Fungal endophytes isolated from healthy cacao plant tissues also displayed *in vitro* antagonism response against major pathogens of cacao, including the WBD (Mejía et al., 2008). The antagonism mode of action was reported as competition for nutrients or antibiosis (Mejía et al., 2008). WBD fungus was though not killed by any of the tested endophytic fungal strains. In another study (Rubini et al., 2005), the endophytic fungus *Gliocladium catenulatum*, isolated from healthy cacao plants, was able to reduce the incidence of WBD in cacao seedlings to 70% in greenhouse conditions. Again, *Moniliophthora perniciosa* was not killed by *Gliocladium catenulatum*. Instead, another endophytic isolate from *Trichoderma stromaticum* parasites internally *Moniliophthora perniciosa*, preventing its reproduction (Samuels et al., 2000; Pomella et al., 2007; Medeiros et al., 2010). No information is presently available as to the molecular basis of this antagonism or the death process associated.

The Brazilian governmental organization CEPLAC (Executive Committee of the Cocoa Farming Plan) has produced and developed a semi-commercial product named Tricovab^®^, that is available to Brazilian farmers on a *by request* basis. This biocontrol agent is multiplied in silos of rice grains, in which surface the fungus multiplies. The local farmers receive the packed dry rice grains and activate the product by making a suspension in water that can be spread directly in the plantation^3^. Despite being an alternative with relatively high efficacy compared to the other methods, it is a very expensive methodology. The high price derives from the very complex and expensive *Trichoderma stromaticum* rice-dependent production system, reason why this method is entirely subsidized by the federal government and supplied as a service from the Brazilian Ministry of Agriculture and developed at its R&D unit CEPLAC^4^ (Silveira, 2013). Additionally, the waste of large amounts of rice diverted from food resource, and the unavailability of the product to local farmers located far from the CEPLAC distribution centers contribute to the impracticability of this solution, reinforcing the urgent need for strategies to control the WBD that are more sustainable. The utilization of antagonistic yeasts as biocontrol agents could be one such case. The possibility of using yeasts for this purpose is very attractive because, unlike filamentous fungi or bacteria, (i) they are ubiquitously found in the phytobiome, displaying a degree of biodiversity that allows the finding of natural and specific antagonisms, (ii) they are GRAS (Generally Recognized as Safe) for humans and animals and therefore safe to manipulate, (iii) they generally promote the wellbeing of the plants, (iv) they are environmental-friendly microorganisms, and (v) they can be cheaply multiplied to very high amounts, particularly in Brazil, in view of the long tradition of yeast-fermentation industries (Figure 1). Two yeast strains were previously shown to strongly antagonize *Moniliophthora perniciosa in vitro*, a *Candida* sp. strain isolated from an organic farm soil and a *Dipodascus capitatus* strain isolated from the jackfruit (Cabral et al., 2009). These or other yeast strains were not further explored in *in field* assays, neither was their mode of action studied.

## Concluding Remarks

The cacao producers from Central and South America, where the WBD is a threat, are being encouraged to substitute their plantations with more resistant cacao plant varieties^5^. This is an expensive and drastic solution for plantations still operating, implicating a production lag phase that not all producers can afford. Moreover, more resistant varieties cannot avoid the incoming of more virulent fungal strains, which appears to be a world-wide trend (Fisher et al., 2012). The use of natural microbial biocides, has in favor not only the health, environment and economic arguments above stated, but also the almost infinite microbial biodiversity available to continuously feed the search for a suitable killer for each emerging or evolving phytopathology. This review calls the attention to this possibility, in particular using phytobiome-originating yeasts, as a sustainable alternative strategy to manage the *Witches’ Broom Disease* of cacao, contributing to ease the associated severe social and economic implications to the life of millions of people.

This is a research area with plenty to explore and a predictable great importance in the field of plant pathology in the next years.

## Author Contributions

PF wrote the manuscript together with FC and CL. All authors read and approved the final manuscript.

## Conflict of Interest Statement

The authors declare that the research was conducted in the absence of any commercial or financial relationships that could be construed as a potential conflict of interest.

## Footnotes

1 www.ICCO.org

2 Available at: https://www.icco.org/about-cocoa/pest-a-diseases.html (accessed February 18, 2019).

3 www.ceplac.gov.br

4 Available at: https://www.servicos.gov.br/servico/comprar-biofungicida-tricovab-para-a-vassoura-de-bruxa-do-cacaueiro (accessed February 18, 2019).

5 Available at: https://www.icco.org/about-cocoa/pest-a-diseases.html (accessed February 18, 2019).
